# Towards a Novel Strategy for Safety, Stability and Driving Dynamics Enhancement During Cornering Manoeuvres in Motorsports Applications

**DOI:** 10.1038/s41598-020-63243-w

**Published:** 2020-04-14

**Authors:** Aditya Roy, Debabrata Dasgupta

**Affiliations:** Department of Mechanical Engineering, Indian Institute of Technology Delhi, New Delhi, India

**Keywords:** Aerospace engineering, Mechanical engineering

## Abstract

With aerodynamics research being primarily focused on drag and lift mitigation strategies till date, engrossed attention has not been received by the event of numerous accidents caused due to negotiation of turns at high speeds by formula and motorsport cars. We present an unexplored and innovative concept of variable side-flap mechanism with a peculiar orientation for introducing three components of forces in the event of turning. Numerical simulations have been performed at two notorious slant angle configurations of the generic ground vehicle body, namely 25° and 35°. In order to analyse the inception of variable forces and flow changes introduced by the flap mechanism, RANS modelling has been performed on nine cases of flap angles from 10° to 90° at six instances of Reynolds numbers between $1.98\times {10}^{6}$ and $2.98\times {10}^{6}$. In addition to the introduction of variable drag and downforce components leading to decrement in braking effort and enhancement in overall stability and safety characteristics based on steering input, our proposal holds potential for improving driving dynamics by imparting mild oversteer characteristics due to a flap-induced vortex. To conclude, a safe regime of flap operation in terms of flap angle based on drag, downforce and oversteering dynamics has also been recommended.

## Introduction

The automotive industry has received a significant amount of attention from researchers worldwide in improving the aerodynamic characteristics of various classes of vehicles, starting from high-speed motorsport cars to heavy load carriers. With the advancement in computational facilities, it has now become possible for simulating the proposed design changes and modifications before arriving at the final design that can be implemented. One of the few objectives of research has been in reducing drag and lift forces associated with the vehicles in motion. This has led to some remarkable aerodynamic designs that we see nowadays in the motorsports industry. Motorsport cars are subject to very high speeds, as a result of which, they experience tremendous amount of lift forces. Front and rear wings are fitted on these cars that generate downforce in order to maintain the overall stability at high speeds^1^. The problem exists in the case when such cars have to negotiate turns and twisty sections on the racetrack. Centrifugal force acting on a car is directly proportional to the square of velocity during turn negotiation, and consequently, drivers need to slow their cars down in order to not lose stability. They rely on the downforce generated by front and rear wings and tire friction for successfully negotiating the turn. While Formula 1 wings and tires generate massive amounts of downforce and friction, respectively, several cases of accidents have been reported till date wherein, the drivers lost control of their vehicles while negotiating sharp bends. While such incidents pose a direct threat to life and property, implementation of design additions specifically focused on turn negotiation and the dynamics involved for mitigation has received scant attention. Motivation can be sought from some of the provoking studies conducted so far.

**Figure 1:**
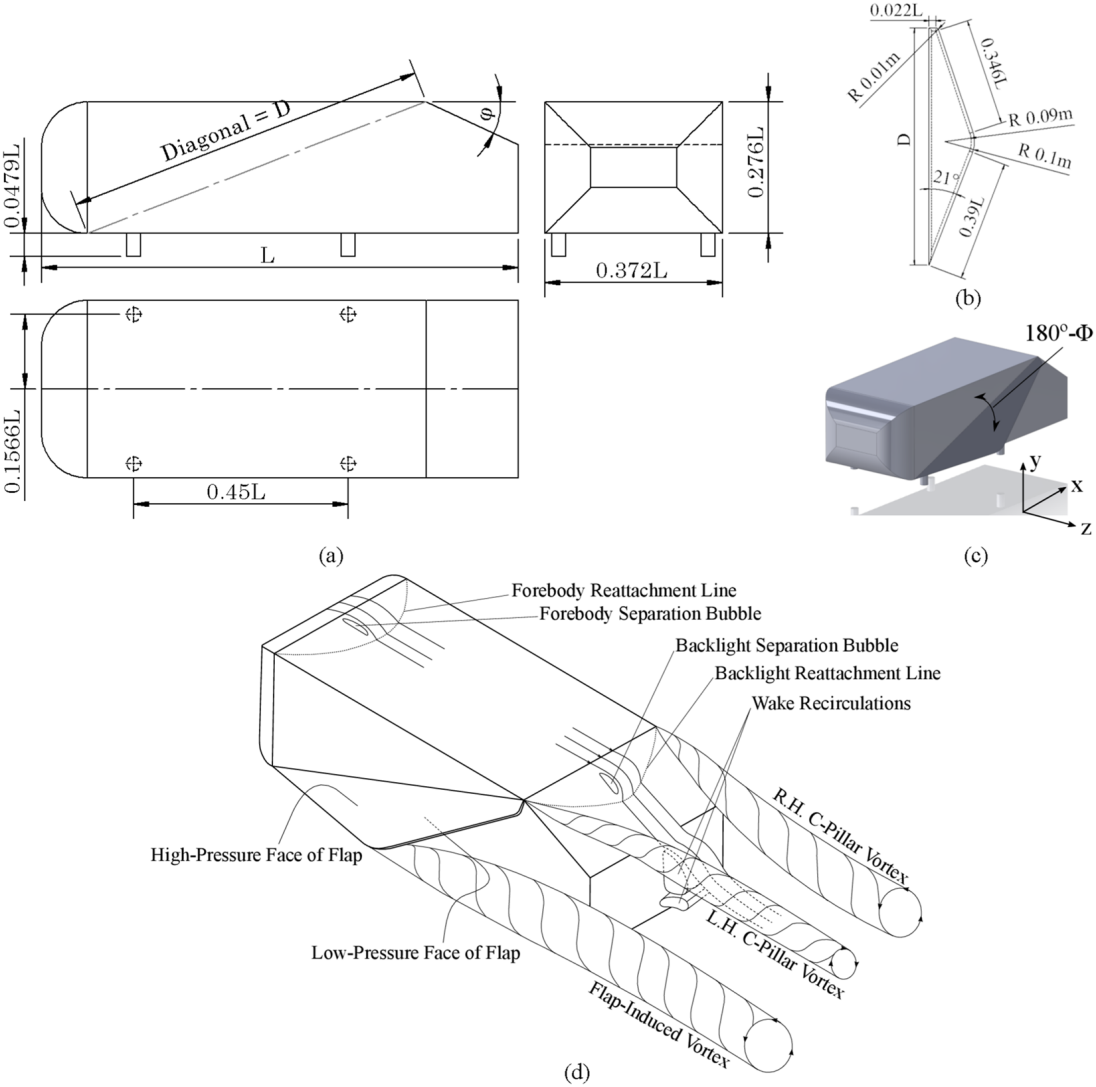
Principal dimensions of (**a**) Ahmed body and (**b**) Flap; (**c**) Assembly of Ahmed body and Flap and; (**d**) Characteristic flow structures. Full-scale tests have been performed by attaching a flap on the generic ground vehicle body, length *L* of which is 1.044 m. The present study has been conducted on two slant angles of 25° and 35°. Flap angle is denoted by φ. The figure indicates wake region recirculations which have been cut into half for better understanding. A high average pressure zone exists on top face of the flap while a low average pressure zone exists on the lower face, as a result of which, a flap-induced vortex gets generated as shown.

Majority of the available literature points towards studies carried out on a simplified bluff body, first introduced by Ahmed *et al*.^2,3^. Now known after the name of the researcher, Ahmed body is a simplified model that represents ground vehicles. Ahmed *et al*.^2,3^ proposed a design of a bluff body which could serve the purpose of studying and analysing the external aerodynamics of ground vehicles. As shown in Fig. 1, the Ahmed body provides a very simplistic, yet intriguing geometry to conduct aerodynamic studies. Effect of slant angle on the wake region, formed at the back of the vehicle, was one of the sole objectives of experimental studies conducted by Ahmed *et al*. Geometry of this body enables the flow to remain attached towards the front, in accordance with the concept of favourable pressure gradient in vehicular aerodynamics, while preserving the scope of studying intricate details of flow over the rear slant, offering relevance to hatchbacks and fastbacks. Although there is a significant amount of literature available that provides insight into the complex flow phenomena taking place on the rear portion of the ground body, some observations regarding the vortical structures being formed may be pointed out here^1,4,5^. Vortical structures resulting due to the presence of a rear slant angle have both spanwise and longitudinal components. The region at and near the centre of rear sloping angle is known as backlight. Longitudinal vortices emanate from the two C-pillars, travelling along the backlight region located at the rear of the vehicle. Besides, due to boundary layer separation taking place at the backlight region, some airstreams rush in for compensating the pressure gradients thus resulting in spanwise wake region vortices. Pressure drag caused due to the rear slant plays a significant role in determining the total drag on Ahmed body^6^. An observation specific to the case with 25° of slant angle is that a detachment zone is formed at the beginning of the backlight region and a subsequent reattachment zone is formed at the surface of the slant before reaching the trailing edge which results in a separation bubble. Previous studies show the complexities involved in capturing the flow phenomena taking place at the backlight region with a slant angle of 25° ^3–5,7–10^. The C-pillar vortices keep becoming more pronounced with an increase in slant angle. However, failure of the separation bubble to reattach was observed at 35° of slant angle, causing the longitudinal vortices to burst. Above a critical angle of 30°, the flow fully detaches over the slant due to the adverse pressure gradient.

Realizing the fact that merit of numerical modelling and simulation lies in the accuracy with which it is able to predict complex flow characteristics taking place in the wake and backlight region, baseline studies conducted in the present work have been benchmarked with the work of Lienhart and Becker^7^ who conducted experimental studies on the Ahmed body in an LSTM low-speed wind tunnel using Hot Wire Anemometry (HWA) and Laser Doppler Anemometry (LDA) measurements.There have been other experimental studies as well^11–14^. The realizable *k-ε* eddy-viscosity turbulence model has been used in the present work for numerically modelling the flow physics associated with the computational geometries of our concept. Detailed insights on the selection of the turbulence model, validation with experimental data and grid dependence study have been presented in the Methods section.

There have been numerous studies on active and passive methods of drag and lift modification strategies having a direct or indirect impact on fuel savings and overall ride stability at high speeds^8,15–23^. Ahmed’s car model serves to be a useful tool for testing innovative concepts before performing tests on real-life cars or models. For the first time, a computational study of an unconventional idea of employing a side-flap spoiler – whose angle from the surface depends on steering input from the driver – for enhancing safety and driving dynamics, and its effect on the external aerodynamics of a ground vehicle body when compared to baseline case is being presented. The objective of this study is to evaluate the potential benefits and flaws of introducing active aerodynamics with the help of the said flap so that it could act as a motivation and starting point for more future studies. When the vehicle is moving on a straight-line path, the flaps remain in the retracted position, and during turn negotiation, they open at an angle based on steering input and the direction (left or right) the car is turning in. Numerical simulations have been performed to model a case where an Ahmed body negotiates a rightward turn. The left flap opens in this case, and the orientation of the same is such that it should induce three components of forces viz., drag force, downforce and inward centripetal force. One of the primary objectives of this work is to demonstrate the introduction of a downward force component which would lead to an increment in stability at winding sections. Secondly, while drag force reduction is one of the prime objectives in vehicle aerodynamics research, an increment in the same at curve negotiations would result in an air resistance, thus reducing the required braking effort. We aim to study how the theoretical proposition of inward force addition by our flap fares when investigated together with the effect of flow changes introduced by the same. Simulations have been performed at length-based Reynolds number values ranging from 1.98 × 10^6^ to 2.98 × 10^6^ for flap angles ranging between 10° and 90° from the surface, in steps of 10°, thus accruing a dataset of 108 cases. Success with the present proposition would prove to be a life-saving innovation which will not only enhance the driver’s safety but would also convalesce the loss of balance and stability at the most winding sections of race circuits. It may also be added that with improvement in road and transit systems globally, this concept has the potential to prove as being beneficial to private and commercial vehicles as well during instances of over-speeding at mild turns on highways, based on the design of the vehicle.

## Computational Geometry

The Ahmed body and flap with all their principal dimensions are depicted in Fig. 1. The flap is attached on the left side of the Ahmed body, along the diagonal connecting the opposite vertices. The objective of this work is to investigate the outcome of attaching one flap each on both sides of a motorsport vehicle, but we have considered only one flap attached on the left-hand side of Ahmed body as at a given time during turn negotiation, only one flap is to open. When the vehicle is turning towards its left-hand side, the right-hand flap will open and vice versa. Initially, a rectangular profile of flap was chosen, and simulations were run for plotting the pressure contours on the surface of the flap. With the aim of cutting maximum possible air, a polygonal profile for the flap was thought upon. Pressure contours plotted on the rectangular profile of flap showed minimum pressure values towards the rear portion near slant region. Thus, the surface area of the flap on the rear portion was reduced subsequently. Aligned with the objective of maximizing pressure force due to flap, the surface area was maximized in regions of high pressure and vice versa, without distorting the geometry of Ahmed body at the retracted position of the flap. This resulted in the present flap geometry from the initial rectangular profile. The angle at which the flap opens or protrudes out from the side is variable. Numerical simulations have been performed for flap angles ranging from 10° to 90° in steps of 10°. Length *L*_*f*_ of the flap is equivalent to diagonal length *D* of Ahmed body. Fillet of radius 0.1 m has been provided on the spanwise corner and 0.01 m radii each on the connection between top and bottom face as well as on the rear corner, in order to deter the generation of strong vortices since while a vortex is expected to form because of an added protrusion from the side, sharp corners would make its generation even stronger.

Computational domain setup and domain discretization play an important role in the accuracy of numerical results. One encounters the terminology of blockage ratio when dealing with closed domain aerodynamics, which refers to the ratio of frontal area of object to the frontal area of wind tunnel. The effect of blockage ratio on results produced in vehicle aerodynamics studies has been well compiled and documented by Keogh *et al*.^24^ who used a blockage ratio of 1% in their study. The domain extents in our work have been set according to the studies of Guilminaeu^25^ and Lanfrit^26^. Length of domain towards the front face and rear face of the geometry is equivalent to approximately 3 *L* and 5 *L* respectively. Length of domain towards the two sides and top of the geometry is equivalent to 2 *L*, which gives a blockage ratio of 1.1%. Turbulence intensity at the inlet of the domain is less than 1%, comparable to that used in the wind tunnel study by Lienhart and Becker^7^.

## Results

### Effect of side-flap addition on drag

**Figure 2:**
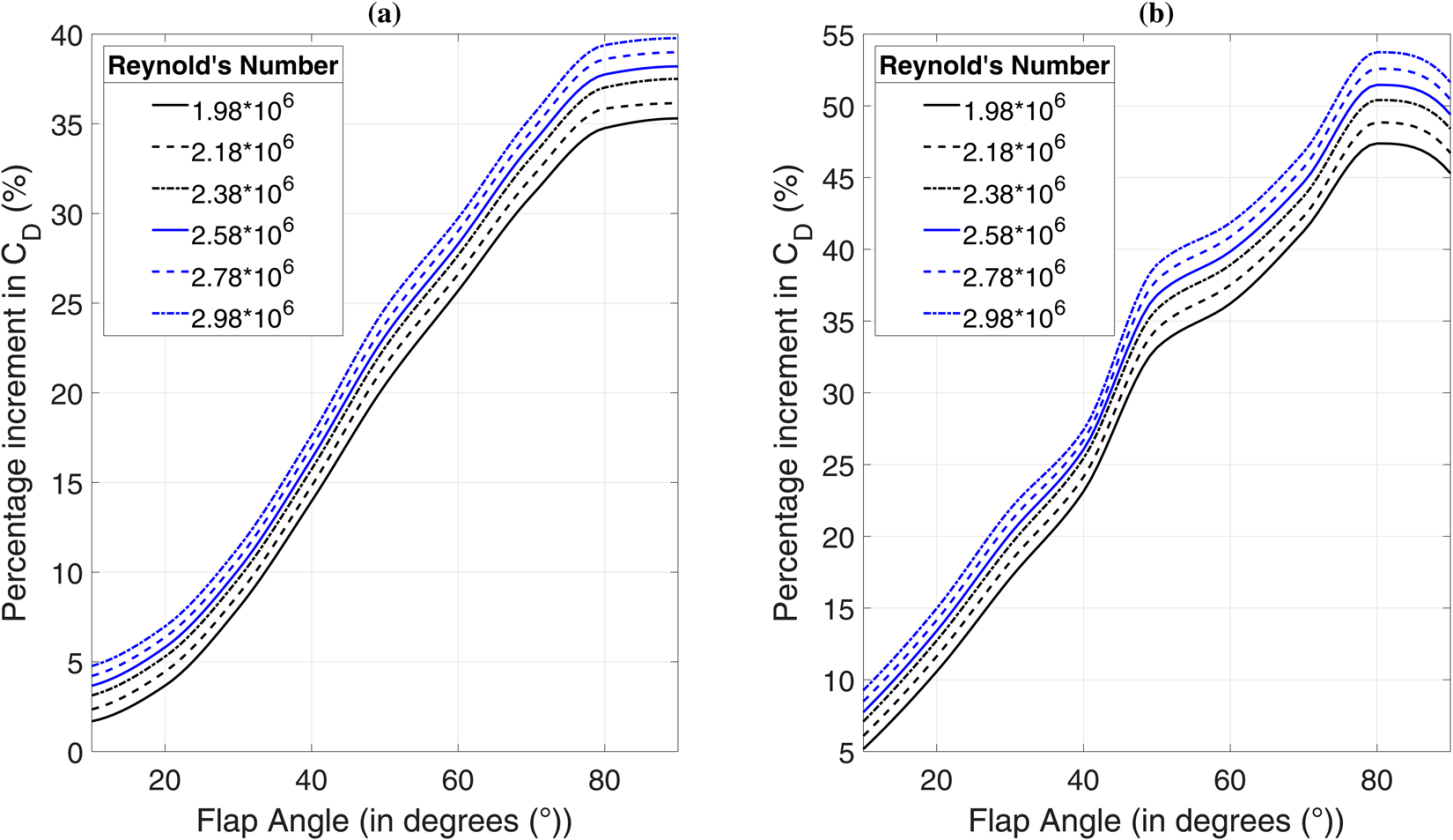
Variation of percentage increment in *C*_*D*_ with flap angle for **(a)** 25° slant and **(b)** 35° slant. In the case of 25° slant, *C*_*D*_ shows a less pronounced variation between flap angles of 10° and 20°. After 20°, *C*_*D*_ shows a steep rise up to 80° post which, the curves nearly become flat. The mentioned variation is a bit different in the case of 35° of slant. *C*_*D*_ shows a steep rise from 10° to about 80° of flap angle and starts diverting from this trend before 80°. A downward trend is seen after 80° until 90° which means that the drag coefficient of 90° flap angle configuration is less than 80° of flap angle. *C*_*D *_for 25° case varies from about 0.298 to 0.401 whereas the same varies from about 0.287 to 0.406 for 35° case. In spite of 35° slant angle case being a low drag configuration, *C*_*D*_ values show a very significant rise with additional side flap. The lowest *C*_*D*_ value at ${\rm{Re}}=2.78\times {10}^{6}$ for 25° (high drag) case is 1.7% higher than the baseline case whereas the lowest *C*_*D*_ value at the same Reynolds number for 35° (low drag) case is 7.6% higher than the baseline case.

Numerical results have been compiled for flap angles ranging from 10° to 90° in steps of 10° at six values of Reynolds numbers between $1.98\,\times {10}^{6}$ and $2.98\times {10}^{6}$, both included. Simulations were performed for both cases of Ahmed body having slant angles of 25° and 35°. Thus, 108 sets of numerical simulations were performed alongside baseline studies for investigation of our concept and finding allied variations in flow phenomena, as represented by *k*-*ε* RANS modelling. Meile *et al*.^14^ investigated the variation in drag coefficient (*C*_*D*_) with Reynolds number (Re) up to 2.78 × 10^6^ and presented a curve fit of their results with those of Ahmed *et al*.^3^ and Bayraktar *et al*.^5^. In the present case, on considering a variation of *C*_*D*_ with Re for various flap angles at the two slant angles separately, a maximum variation of 1% was observed within the given range of Re. As per the terminology defined by Roshko^27^ and comparing our results with the curve fit presented by Meile *et al*.^14^, our investigated cases with realizable *k-ε* RANS turbulence modelling happen to lie in the transcritical range whereas this range of Re falls in the transitional regime for a simple Ahmed body without side flaps (for more information, please see Supplementary Information). Plots of percentage increment in *C*_*D*_ and its variation with flap angle are shown in Fig. 2 for both the slant angles. Flap angle has a direct relationship with wind resistance. Greater the angle, greater would be the frontal area of the vehicle resulting in a higher amount of wind resistance. If we only consider the aspect of frontal area and its effect on wind resistance, we should expect a linear curve between *C*_*D*_ and Re because drag coefficient is a measure of wind resistance possessed by a vehicular body. Here we instead see a non-linear relationship between *C*_*D *_and Re. It may be concluded from this ambiguity in *C*_*D*_ and Re relationship that increase in the frontal area due to the addition of side flap is not the sole cause of drag modification. There are other factors at play that govern this relationship. It would be seen in the upcoming paragraphs that vortex generation due to hindrance in the flow from side flap causes significant changes in the flow phenomena taking place at backlight and wake region which contribute to the non-linear relationship shown in Fig. 2. Drag increment is nearly equal for flap angles of 80° and 90° in the 25° slant case. On the other hand in case of 35° of slant angle, percentage drag increment at 90° of flap angle is less than that at 80° of flap angle. It can thus be said for both these cases that increasing the flap angle beyond 80° does not serve any benefit considering the factor of braking effort decrement (for a similar explanation considering drag force, please see Supplementary Information).

It is worthwhile to discuss the advantages that drag addition brings about in the event of turn negotiation. We have already seen in the previous sections how research in vehicle aerodynamics has been focused towards drag reduction and making the designs more streamlined. While environmental considerations and stringent norms imposed by authorities support this direction of research, speaking of which, new drag reduction measures are need of the hour, turning manoeuvres is where variable drag addition might help. This could be explained as follows. When a vehicle travelling at high speed has to negotiate a winding section, the driver first applies brakes to slow down the car to a safe limit of speed depending on various factors such as banking angle, car weight and centre of gravity. The reason behind doing this is to maintain stability considering the centrifugal force trying to push the car outwards. As soon as the turn is made, the driver again accelerates and moves forward. The intensity of this problem heightens when we consider the case of formula cars that travel at straight-line speeds of more than 300 kmph and slow down to around 100 to 150 kmph at sharp hairpin bends, which warrants a significant amount of braking effort. In other words, it is desirable to add resistance components right before an event of turn negotiation. From Fig. 2, it is evident that the overall drag coefficient increases with an increase in flap angle. Here we propose a variable flap mechanism according to the steering input provided by the driver. Steering input in the event of a very sharp hairpin bend would be more than that in the event of a mild turning section. Greater the steering input, higher would be the side flap angle resulting in higher wind resistance. Increased wind resistance, its extent depending on the effective radius of turn, would result in reduced braking effort on the part of driver. As soon as the vehicle has exited the twisty section with its steering pointing straight, the side flap would retract, and the car would exhibit its usual aerodynamic characteristics. This would especially be of advantage to formula car drivers who always try to reduce their cars’ brake usage. We are familiar with the amount of heat generation involved at the brake pads and discs of such cars thus continually reducing their life. Variable side flap mechanism, being proposed here, would result in an increase in service life of the brake pads along with an enhancement in safety.

### Effect of side-flap addition on lift

**Figure 3:**
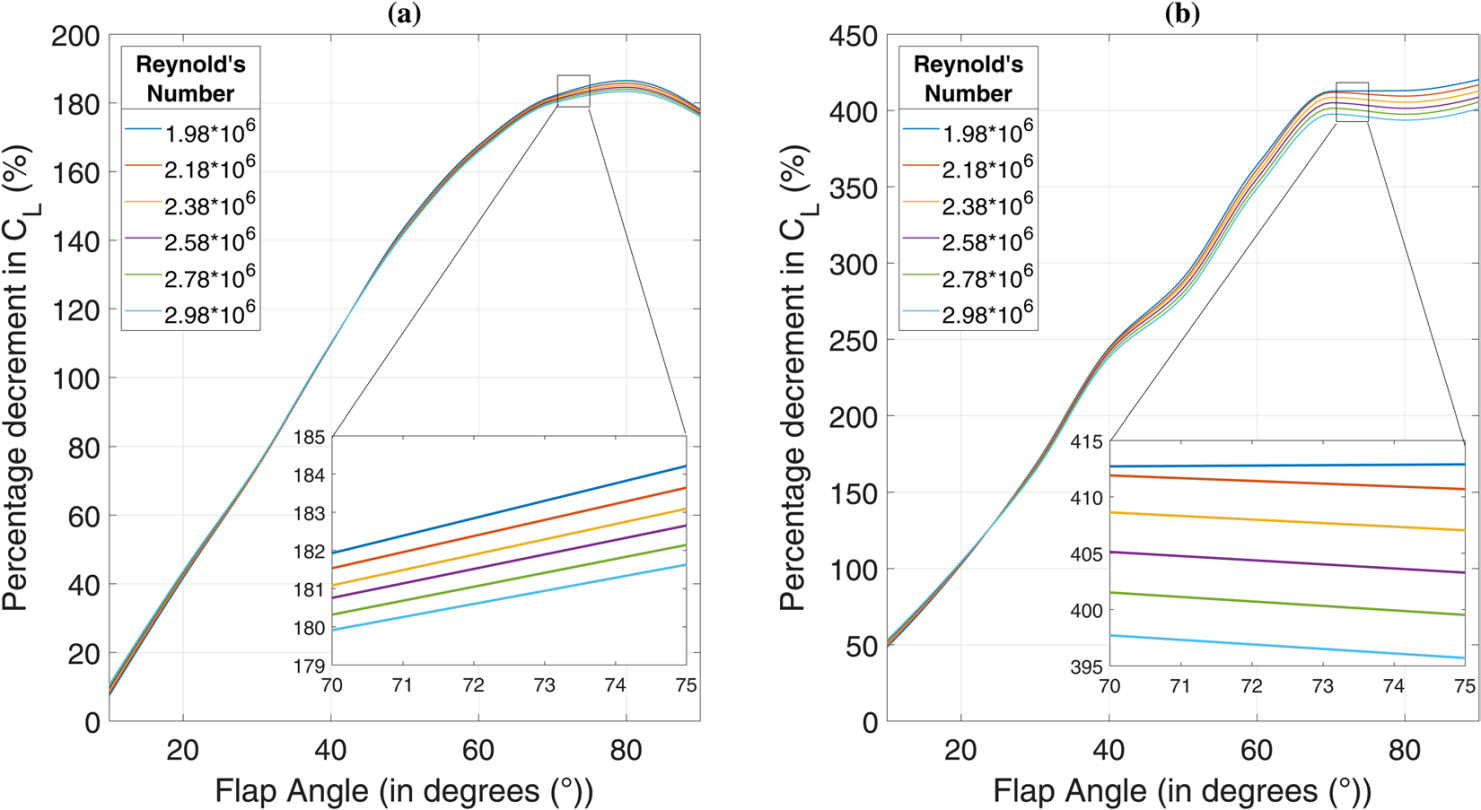
Variation of percentage decrement in *C*_*L*_ with flap angle for **(a)** 25° slant and **(b)** 35° slant.The highest negative value of lift coefficient in the case of 25° slant is of the order of −0.29 whereas the same in case of 35° slant is of the order of −0.47. Percentage of overall decrement in lift coefficient crosses the 100% mark just before 40° of flap angle in the 25° case whereas the same event happens right before 20° of flap angle in the 35° case. Moving beyond 100% decrement in *C*_*L*_ means that there is a net positive downward force acting on the vehicle, and there is no possibility of instability due to vertical force components. An overall negative lift coefficient at target speeds would enhance the stability of a vehicle by several folds during the event of turn negotiation. There is another important point of observation that was drawn from the solution dataset. In case of 25° slant, percentage decrement in *C*_*L*_shows an increasing trend for respective flap angles as we move up the Re range for flap angle values of 10°, 20° and 30° till the Re value of $2.78\times {10}^{6}$ for 40° of flap angle after which, the values show a decreasing trend for their respective flap angles (50°, 60°, 70°, 80° and 90°). This critical configuration in case of 35° slant case is at ${\rm{Re}}=1.98\times {10}^{6}$ for flap angle of 30°.

The decrement in overall lift coefficient (*C*_*L*_) of a vehicle is one of the sole objectives that this study holds. Figure 3 presents an assessment of the lift characteristics possessed by a generic ground vehicle body with flaps attached, for both the slant angles. The flap acts as a hindrance in the path of free-flowing air because of which, the wind pressure is exerted on the same. Theoretically, three components of this resultant force may be drawn, one of which represents downforce. Therefore, the lift force being generated by the air flow on the vehicle body is expected to decrease. In practice, it is seen that the pressure force acting on flap surface creates a pressure gradient, with positive pressure acting on the top surface and negative pressure being generated as a result on the bottom surface. Incompressible flow physics governs the flow of air from the high-pressure side to the low-pressure side, and this results in two important outcomes. Firstly, there is a net increment in the downforce being generated due to the pressure gradient formed depending on the flap angle and secondly, there is inception of a side vortex being generated. Effect of the same on dynamics during cornering will be explained in upcoming paragraphs. The variation of *C*_*L*_ with Re suggests that there is a net decrement in lift coefficient right from the case of 10° of flap angle for both slant angle configurations. Figure 3 shows that there is a higher percentage of decrease in *C*_*L*_ in 35° slant case as compared to the 25° slant case. This is primarily because of the fact that 25° baseline case has a high lift coefficient of the order of 0.35 whereas 35° baseline case has a reasonably low lift coefficient of the order of 0.01^14^.

As shown in Fig. 3, a decrease in lift coefficient directly translates to a decrease in lift force experienced by the vehicle and has a direct impact on its stability during cornering manoeuvres (for variation of increment in downforce with flap angle, please see Fig. S4 in Supplementary Information). We know that centrifugal force acting on the vehicle tries to push it outwards as a result of which, there is weight transfer from the centre of gravity (CG). Considering the case of a baseline ground vehicle body negotiating a right turn, the weight transfer would be on the left outward direction. Due to the presence of a flap on the left-hand side, there would be a downforce increment. This downforce would not be acting along the line of CG of the vehicle but along an axis shifted on the left of CG towards the flap. There would be more weight transfer on the two wheels on the left-hand side and thus, a higher amount of tire friction, which would have a tendency of acting inwards due to centrifugal force trying to push the vehicle outwards. The extra downforce, in a way, acts on wheels that need it the most during such an event. This would lead to an enhancement in the stability during turn negotiations. In the case of 25° of slant angle, the downforce values increase steadily up to 70° of flap angle. Between 70° and 80°, while there is an enhancement, it is not as pronounced as previous angles. There is maximum downforce increment at 80° of flap angle after which the curve follows a decreasing trend. As a result, there is no point increasing the flap angle beyond 80°. On the other hand, downforce enhancement is steady till 60° of flap angle in 35° slant case after which it starts slowing down. The values are nearly equal between 70° and 80° after which there is a slight improvement until 90°. We may thus conclude that increasing the flap angle beyond 70° would serve little benefit, thinking from the perspective of stability enhancement due to downforce increment.

### Effect of side-flap addition on Z-force

**Figure 4:**
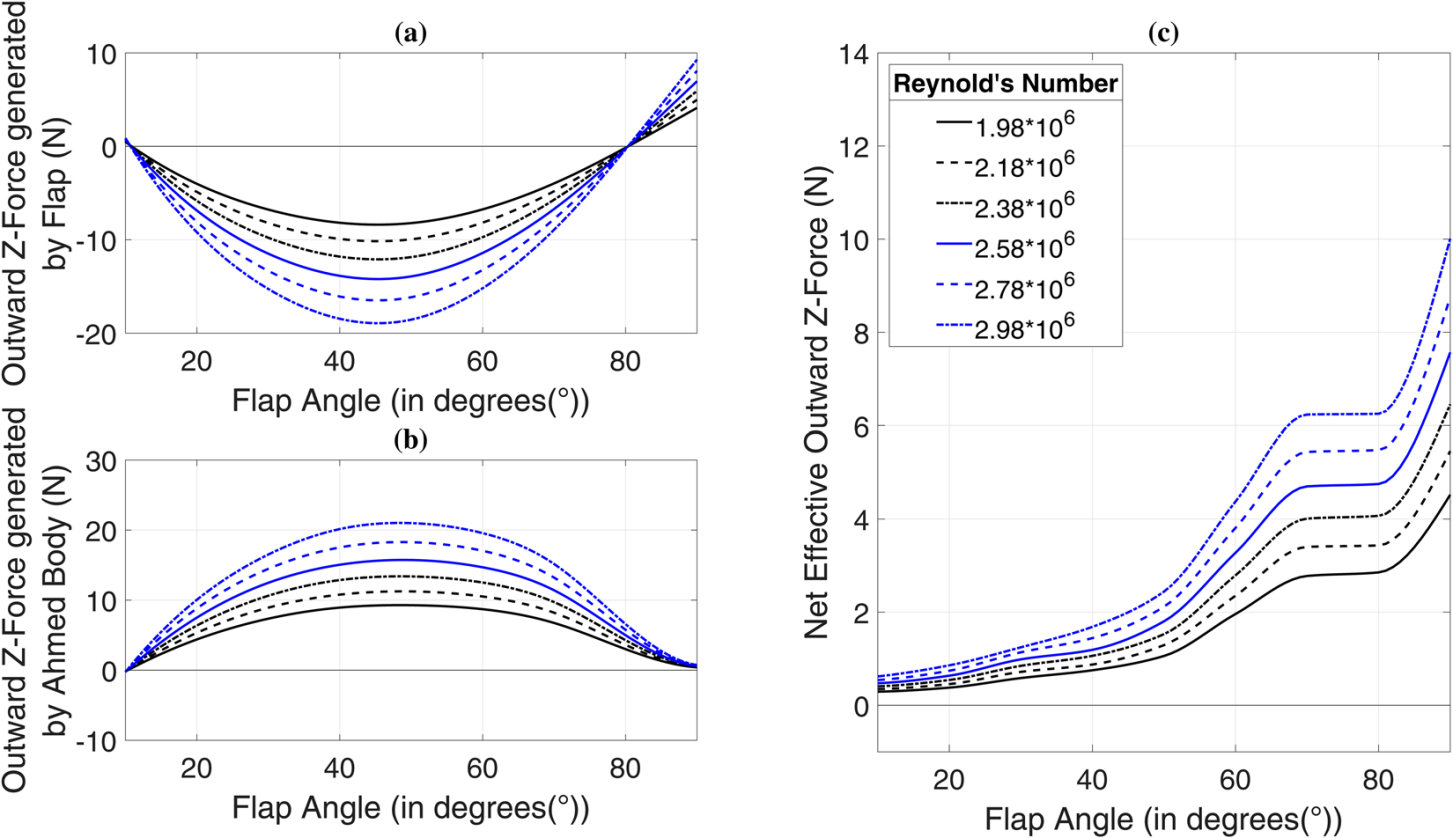
Outward Z-force generated by (**a**) flap, (**b**) Ahmed body and (**c**) net effective outward Z-force versus flap angle for 35° of slant angle. Speaking first about 35° slant angle case, initially at 10° of flap angle, a certain amount of outward centrifugal force is generated by the flap at all Re values. The forces become strictly centripetal after 10°, post which they follow a steady rise. Centripetal forces increase up to 40° of flap angle and reach a point of maxima between 40° and 50°. The inward force generated starts to fall again from 50° up to 80°. The curves cross zero-line immediately after 80°, and the flap again starts generating outward force till our investigated case of 90°. Curves of inward force generated by flap and outward force generated by Ahmed body follow quite a similar trend. The outward force of the Ahmed body increases up to a point between 40° and 50° of flap angle and decreases thereafter. The cumulative effect of both the forces shown in Fig. 4(a,b) results in a net outward force as shown in Fig. 4(c). The increase in net outward force shows a faster rate between 20° and 50° as compared to that between 10° and 20°. The curves show a high rate of increase between 50° and 70° after which they almost remain constant up to 80°. A rapid increment is exhibited between 80° and 90°.

**Figure 5:**
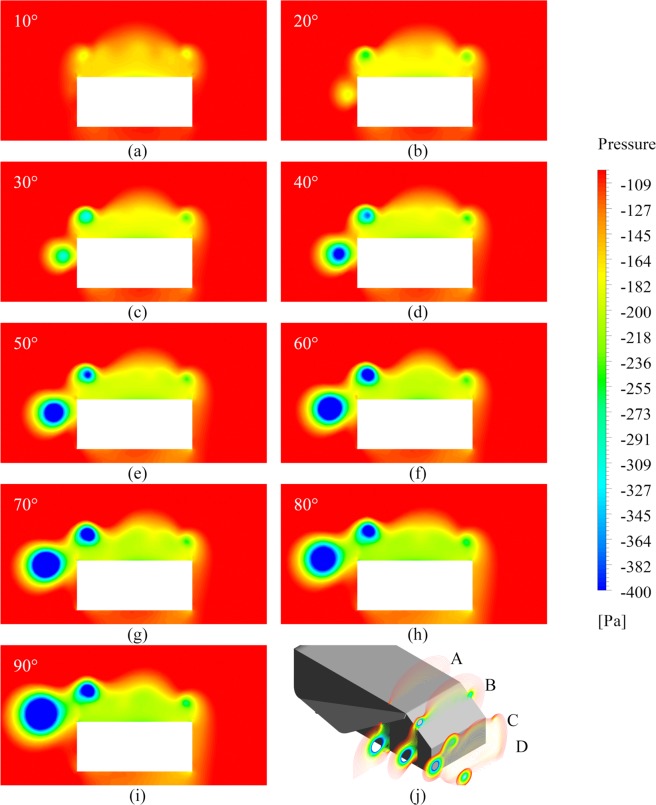
Pressure contours of 35° slant configuration possessing flap angles ranging from (**a**) 10° to (**i**) 90° and (**j**) development of low pressure zones due to vortex interaction at planes A: −300mm, B: −100mm, C: 100 mm and D: 300 mm along x-axis from the rear face of the body at ${\rm{Re}}=2.78\times {10}^{6}$. It is worthwhile to discuss about the pressure contours mapped on YZ plane at a distance 1.034 m from the front of the Ahmed body, shown in Fig. 5 for all flap angles at Reynolds number of $2.78\times {10}^{6}$ for 35° of slant angle. Initially, at 10°, the effect of FIV is negligible, and the right-hand C-pillar vortex (RHCPV) occupies a more extensive spread than the LHCPV thus resulting in an inward force being produced by Ahmed body as observed from the brief negative force values in Fig. 4(b). Such a scenario changes as we move past to 20° case. The FIV starts building up and is less negative than the LHCPV (for a depiction of vortex interaction with the help of path lines and vorticity contours, please see Fig. S7 in Supplementary Information). On comparing the relative strengths and sizes of the LHCPVs and FIVs on the chosen plane, we may comment that both the factors are more significant in the LHCPVs for 10° and 20° while the case of 30° exhibits similar characteristics for both FIV and LHCPV. From 40° until 70°, the strength and size of FIVs are more as compared to LHCPV. The cases of 80° and 90° exhibit greater strength of LHCPVs and greater size of FIVs. Speaking of the relative strengths of vortices formed in two successive cases, we find that the FIV formed in a particular case has higher negative vortex core pressure than its preceding angle case until reaching 70°. There is a substantial drop in FIV core pressure between 70° and 80° which again increases from 80° to 90°. A correlation of the same may be drawn with Fig. 4(c).

We have discussed two out of three force components generated due to the attachment of the side flap. An inward centripetal component is additionally generated by the flap, as shown in Fig. 4(a) for 35° of slant angle. Due to hindrance caused in the path of free-flowing air by the flap, a pressure gradient is generated on the flap surfaces because of which, a vortex is formed as discussed above. The vortex strength depends on more than one factor such as flap angle and Reynolds number. The left-hand C-pillar vortex (LHCPV) generated above the slant undergoes a change in structure due to interaction with the newly generated flap-induced vortex (FIV). Negative pressure zone formed due to flap-induced vortex tries to ‘pull’ the ground vehicle body, as can be understood from the plot of outward force generated solely by Ahmed body, shown in Fig. 4b. Variation of net outward force generated by the combination of Ahmed body and flap finds relevance with the pressure contours shown in Fig. 5. The inward force generated by flap increases from 10° to its point of maxima because of the fact that the amount of hindrance created in path of free-flowing air increases as we increase the flap angle. This can also be understood from the increment in the frontal area with an increase in flap angle. As a result, all three force components, namely drag force (Fig. 2(b)), downforce (Fig. 3(b)) and inward force due to flap (Fig. 4(a)) increase steadily up to the point of maxima (Fig. 4(a)). A decrement in inward force component after maxima point is accompanied with a high increment in overall drag force as can be seen in Fig. 2(b) and a low rate of increase in downforce as can be observed from Fig. 3(b). After surpassing about 50° of flap angle, the downforce starts increasing rapidly until 70°, but drag keeps on increasing up to 80°. The region between 80° and 90° is dominated by net outward force, as can be understood from its rapidly increasing trend. Inward forces generated by flap start to decrease after the point of maxima due to the dominance of flap induced drag force and downforce components. Figure 5 shows pressure contours plotted on a plane 1.034 meters from the front face of the vehicle and expands on reasons behind variation of curves depicted in Fig. 4.

**Figure 6:**
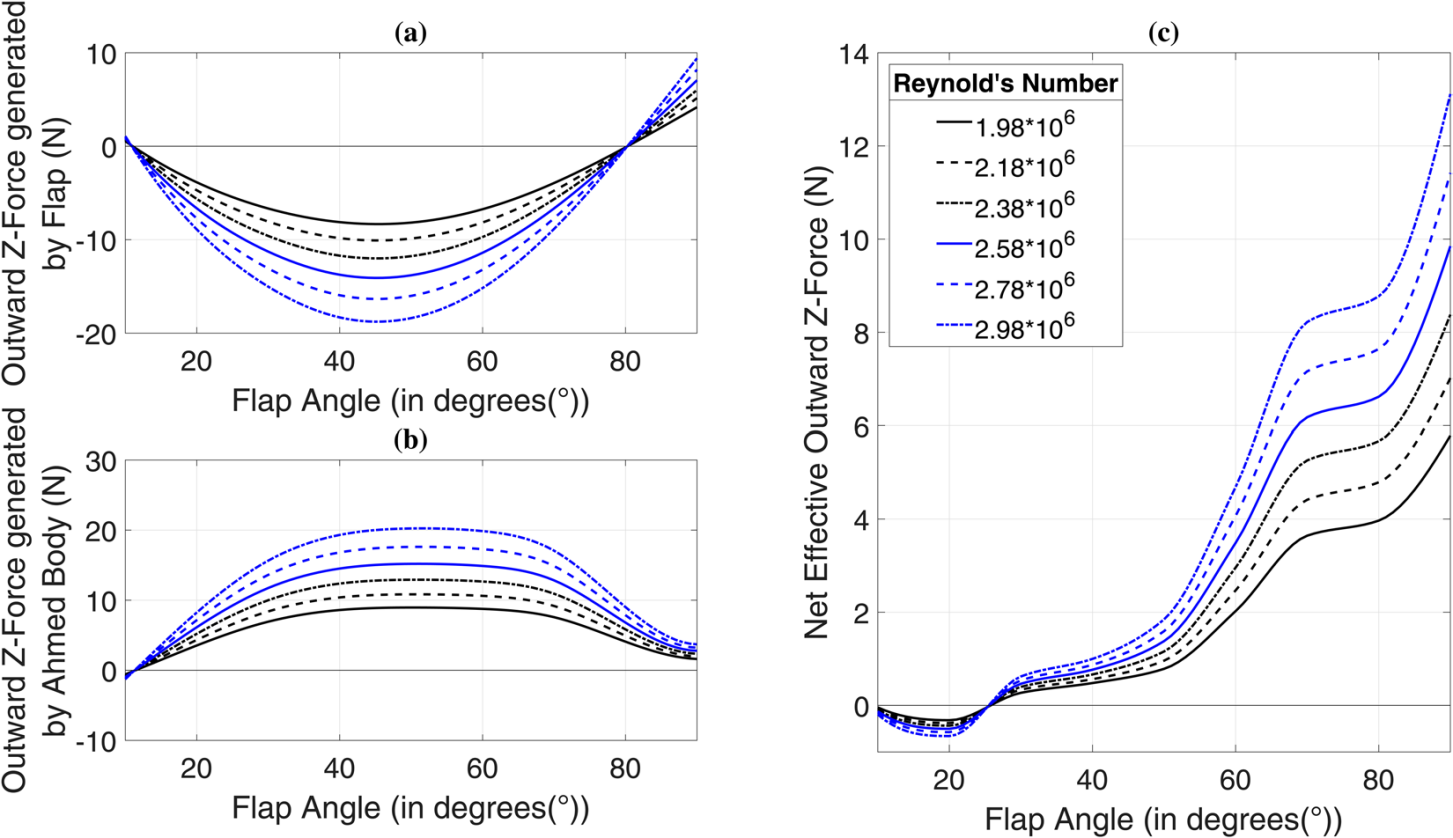
Outward Z-force generated by (**a**) flap, (**b**) Ahmed body and (**c**) net effective outward Z-force versus flap angle for 25° of slant angle.

**Figure 7:**
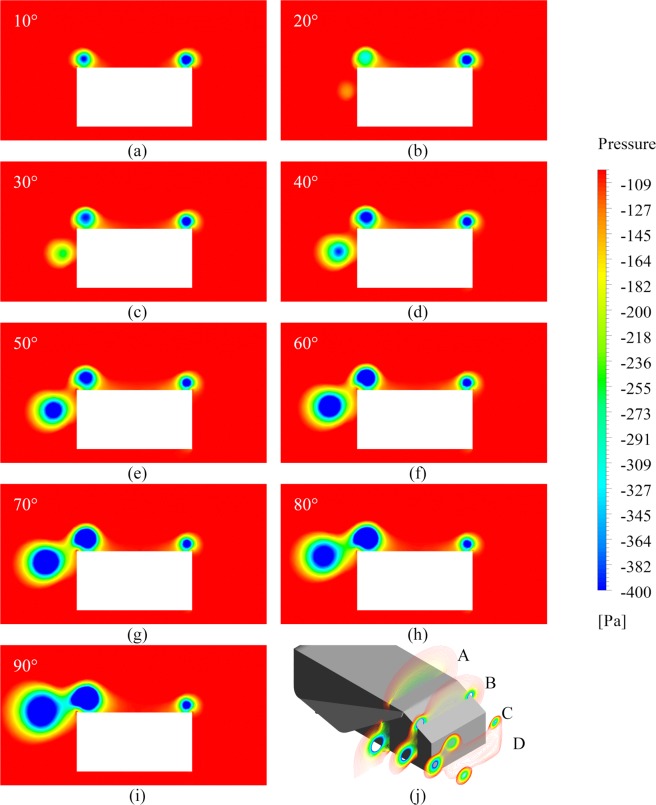
Pressure contours of 25° slant configuration possessing flap angles ranging from (**a**) 10° to (**i**) 90° and (**j**) development of low pressure zones due to vortex interaction at planes A: −300mm, B: −100mm, C: 100 mm and D: 300 mm along x-axis from the rear face of the body at ${\rm{Re}}=2.78\times {10}^{6}$. The first and foremost observation that can be made from these contours is that a zone of separated flow, as seen in 35° slant case above backlight region, is not present since flow separation at the beginning of backlight region is followed by reattachment before the end of backlight slant. The effect of FIV is negligible in the case of 10°, and the RHCPV is stronger than LHCPV. The difference between negative pressures produced by LHCPV and RHCPV in the case of 25° of slant is more than that in the case of 35°. This is why a net inward force component can be seen in Fig. 6(c) as opposed to a net outward component in Fig. 4(c) at 10° of flap angle. The FIV builds up after 10°. Interaction of the air flow starts taking place between FIV and LHCPV, but there is a large amount of difference between the negative pressures of LHCPV and RHCPV thus resulting in the inward force values at 20°. The FIV keeps on getting stronger as we increase the flap angle, except the transition from 70° to 80°. Strength of vortices in terms of negative vortex core pressure is more in the LHCPV for all flap angle cases except the case of 50°. The size of LHCPV is larger than FIV from 10° to 30° whereas the size of FIV is larger than LHCPV for all angles between 40° and 90°. These characteristics act as additional points of difference from 35° slant case.

Observations along a similar direction can be made for the case of 25° slant angle. The variation of inward centripetal force generated by flap with flap angle, as shown in Fig. 6(a) is similar to that in the case of 35° of slant (Fig. 4(a)). Initially, an outward centrifugal force is generated by the flap at 10° of flap angle which soon changes into an inward centripetal force component. Maxima is achieved at nearly 50° following which, the force components drop. At the point of maxima, the factor of dominance is played by drag force component which may be seen to rise in Fig. 2(a), similar to what was observed in case of 35° of slant angle. While the talked-about rise in the drag was particularly pronounced in 35° slant case, it is rather subtle in the 25° counterpart. The transition from inward force component generation to outward type, similar to that shown in Fig. 4(a) is observed just after 80°. It may be noted from Figs. 4(a) and 6(a) that the numerical values of force components produced in both the slant angle cases are almost identical. While the plot of outward force generated by Ahmed body against flap angle (Fig. 6(b)) exhibits a similar trend as well with the case of 35°, there is some variation in the trend of net effective outward force when we consider the cumulative effect of flap and Ahmed body, as may be observed in Fig. 6(c). Net outward force is negative for 10° and 20° of flap angle which means that there is an effective inward centripetal component of force acting on the vehicle body up to a point between 20° and 30°. There is a significant jump in the outward force values from 20° to 30°, post which, the rise is steady. The curves may be seen to shoot up after 50° till 70° followed by a flat region between 70° and 80°. There is a high rate of increase yet again from 80° to 90°. The amount of variation shown for each of the flap angles increases with Reynolds number. Figure 7 presents pressure contours mapped on a plane 1.034 m from the front face of the vehicle and elucidates the variation in curves shown in Fig. 6. For an account on velocity distribution along a horizontal plane at various distances from the ground, please refer to Fig. S5 and Fig. S6 in Supplementary Information.

**Figure 8:**
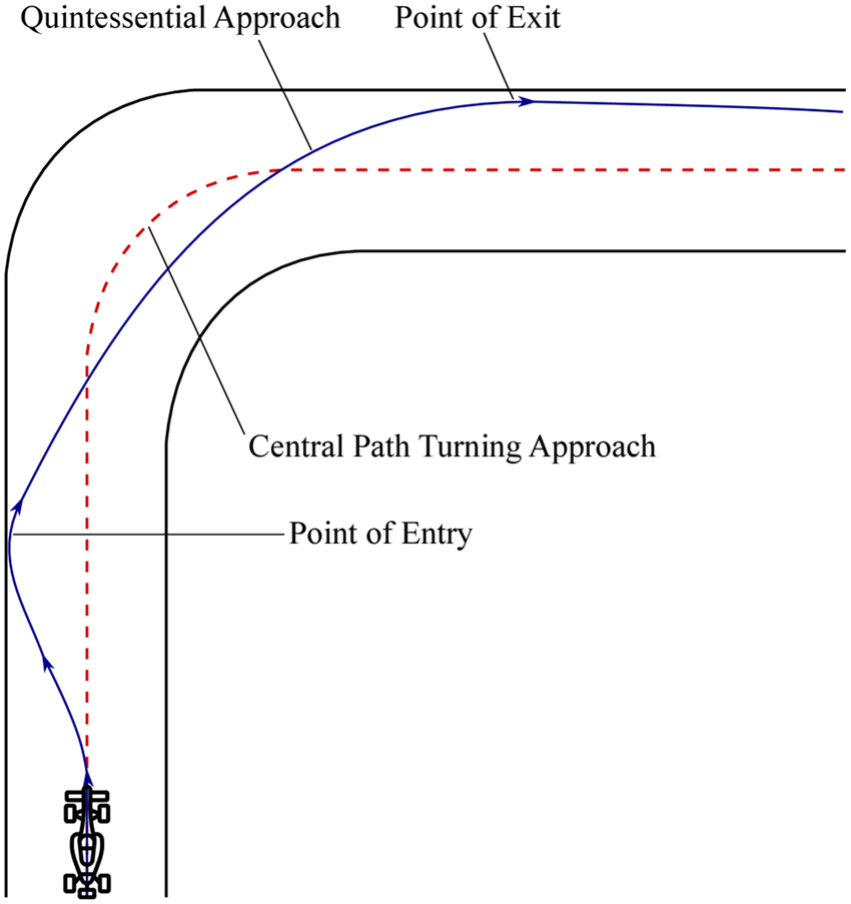
High-speed turning strategy. Experienced drivers never follow a central path turning approach because of the high speed at which they travel. Instead, they follow a path, as highlighted by the quintessential approach in Fig. 8. It is essential to understand the way a sharp turn is made in order to appreciate the advantage of a slightly positive net Z-force. Each millisecond is critical in F1, and thus, drivers tend to waste as minimal time as possible at turns. Following the central path turning approach would require a greater amount of steering input resulting in heavy braking from very high triple-digit speeds. For avoiding the factors of loss of time and improving stability characteristics, the drivers reduce their speeds by a small amount and pass through the point of entry. Some amount of oversteer is desirable at this point, and drivers make the right use of their steering-brake combination in order to facilitate this. Drivers never want excessive oversteer because that would deviate them from their turning approach and possibly lead to an accident. A little bit of oversteer helps the drivers make their rear wheels spin more and record higher speeds during turns, ultimately resulting in lesser loss of time. As soon as they partially drift their way to exit point, they counter steer in order to do away with the oversteer and then accelerate again on the straight path.

It may be seen that while there is the inception of flap generated inward centripetal force, the net effective Z-force acts in the outward centrifugal direction. Apparently, this might seem to be a counteracting factor to our proposal, but in reality, it acts as a factor for enhancing the dynamics during turning. This may be explained with the help of Fig. 8, which shows the approach of an F1 driver negotiating a sharp bend. Modern-day F1 chasses are made to oversteer keeping in mind the event of turn negotiation at high speeds^28^. When fitted on to the rear portion of an F1 car, the side spoiler would introduce an FIV as seen above. According to Figs. 5(j) and 7(j), the built-up FIV would interact with the LHCPV to give rise to low pressure zones based on the flap angle (Figs. 5(a–i) and 7(a–i)). This leads to the net outward Z-force as seen in Figs. 4(c) and 6(c). On correlating these figures, it would be evident that the talked about outward force acts near and beyond the C-pillar. Now, if the vehicle is turning towards its right-hand side, the explained effects would attract the rear portion of the vehicle towards left, thus facilitating potential oversteer. It can thus be said that the Z-force component enhances vehicular driving dynamics while X and Y-force components enhance safety and stability characteristics during turns. It would further be concluded that there exists a safe region for flap operation in terms of flap angle keeping in mind the drag, downforce and oversteer factors.

### Optimum range of flap-operation

Safe zones of operation in terms of flap angle can be identified for the two slant angle configurations based on drag, lift and Z-force characteristics. In the case of 35° of slant, the maximum flap angle generated inward force is obtained between 40° and 50° of flap angle. Post this, the flap generated inward force not only decreases, but the net effective outward Z-force also shoots up (Fig. 4c). As mentioned above, some amount of oversteer is beneficial for drivers during turn negotiation, but too much of oversteer may cost a toll on their safety. Having said that, the maximum flap angle is to be limited to 50° despite the increment shown by drag forces and downforces at higher angles. Based on the results of discrete values of flap angles studied, the prescribed range of operation for slant angle of 35° is between 10° and 50° of flap angle. The case of 25° slant is a bit more complicated. Maximum flap angle in this case as well, is to be limited to 50° because of the same reason as mentioned for 35° configuration. The lower limit would be dependent on manoeuvring expertise of the driver. It may be set to 10° for a highly experienced driver. In this case, the vehicle would be able to take stability advantage of added net effective inward Z-force at mild turns served by flap angle up to between 20° and 30°. At sharper turns served by flap angle beyond this point, the driver would be able to benefit from oversteering characteristics possessed by the vehicle up to 50° of flap angle. With the factor of downforce enhancement at turns, the vehicle would be able to negotiate both, mild and sharp turns at higher speeds within the mentioned safety regime of flap operation.

## Discussion

Introducing three components of forces with the help of a variable side flap mechanism based on steering input is an unexplored concept in the field of vehicle aerodynamics, and the present work is among the first of its kind to present an analysis of effects and repercussions of the same by taking cues from drag and lift characteristics and interaction of flap-induced and C-pillar vortices. Numerical simulations using RANS modelling were performed on two slant angles of the generic ground vehicle body at six values of Reynolds numbers. In order to further our understanding of changes in flow phenomena, a total of nine flap angles were studied for each of the Reynolds numbers and slant angles. While the application of the variable side flap concept would yield different results based on the geometry of the vehicle it is being attached to (and also, the geometry of the flap), a study considering two slant angles cases of the Ahmed body provided grounds for concept validation and proof of potential. Drag coefficients were seen to increase with flap angle meaning that there is a greater amount of wind resistance-generated braking at tighter turns, correlating with an equivalent decrement in braking effort on driver’s part and enhancing the aspect of safety at the same time. Lift coefficients were seen to decrease with increase in flap angle. This resulted in an increment in downforce acting on the vehicle body with a direct impact on stability at bending sections. Interestingly due to weight transfer during turns, the downforce increment would result in more friction at outer wheels which as well, need it the most in order to maintain stability. While the flap was being able to generate an inward Z-force component, the investigated net effective Z-force component of the Ahmed body with flap was pointing towards the outward centrifugal direction due to flap-induced vortex generation. This factor holds significant potential for improving driving dynamics during cornering due to the oversteering approach applied by experienced drivers of motorsport cars. The tendency to oversteer would as well be along the lines of efforts made by modern motorsport teams in intentionally incorporating oversteering characteristics in their chasses.

## Methods

A plethora of numerical studies have been performed on the Ahmed body, some of which investigate the accuracy of various turbulence models while some investigate the changes in flow phenomena induced by addition of active or passive flow control devices. Reynolds averaged Navier Stokes (RANS) modelling is one of the widely used turbulence modelling techniques by researchers, primarily because of the low computational costs involved. At the same time, it exhibits compromised accuracy in case of highly separated flows, but a calculation of the ratio between cost and accuracy provides motivation to use this technique for initial investigations of a concept in hand. Guilminaeu^25^ performed a comparative analysis of the performance of modelling techniques on an Ahmed body with 25° and 35° of slant angles respectively. It was reported that RANS modelling is fairly proficient in capturing all the flow properties of external flow past an Ahmed body having a slant angle of 35° due to the absence of a characteristic detachment and reattachment bubble in the backlight region. However, owing to the factor of presence of the talked about separation bubble in case of 25° of slant angle, all the eddy-viscosity turbulence models suffered in accurately capturing the same, although the drag coefficients were in satisfactory agreement with the experimental values of Lienhart and Becker^7^. Large-eddy simulations (LES) were found to be most nearly predicting the results when compared with experimental observations. Kranjovic and Davidson^4^ were among the first researchers to perform large eddy simulation on the peculiar case of Ahmed body having 25° of slant angle. Although LES was able to capture the complex flow phenomena, it did so at steep computational costs. Following the superior results provided by LES technique, several researchers have focused on a variety of nuances of complex turbulent flows being predicted by LES and its variations^24,29–31^. Realizing the high computational costs involved with LES, relatively less expensive, yet accurate modelling techniques have evolved and been researched upon in recent times which involve the use of partially averaged Navier Stokes (PANS) modelling and hybrid-RANS modelling^24,32–34^. Although such techniques present an advantage over LES in terms of computational cost-effectiveness, RANS modelling performs fairly in capturing the C-pillar vortices and wake region recirculations accurately, as seen in the past, alongside satisfactory prediction of drag and lift coefficients, which could be used for understanding a novel concept in hand within the lowest simulation runtime.

Two categories of the simulation were performed in this study, one using the baseline case of Ahmed body without flaps and the other involving flaps fixed on Ahmed body, both for 25° and 35° of slant angles. Hybrid meshing technique was used in which the domain was discretized using hexahedral elements in the region of boundary layers and ground, and polyhedral elements elsewhere. Using polyhedral elements proved to be advantageous as they reduced the number of elements thus ensuring faster solution runtime, alongside providing faster convergence in fewer iterations. We monitored the closeness of drag coefficients obtained from our numerical model setup with experimental ones and found a disagreement of about 10% with the coarsest grid. As a result, grid refinements were performed for which three bodies of influence were introduced. One enclosed the car model with extents of 1 *L* to the rear, 0.5 *L *to the front, height of 0.8 *L* from ground and 0.45 *L* towards the sides, where *L* denotes car length. The other two bounding boxes provided a higher degree of refinement than the previous one in the wake and underbody regions. Element sizes near the wall were set according to y+ criterion of more than 30 up to 150 and non-equilibrium wall function approach was applied for modelling near-wall flow phenomena. While many of the numerical studies conducted on Ahmed body comply with y+ values being in the viscous sublayer of the wall, a recent work by Tian *et al*.^23^ highlights the use of y+ values in log-law region for obtaining accurate drag predictions.

Literature suggests that RANS modelling successfully predicts flow phenomena for the case of 35° of slant angle but struggles in doing so for the 25° case. Due to the low computational costs involved in eddy viscosity RANS modelling, when compared with large-eddy simulations, we decided to check the accuracy of drag coefficient prediction of baseline case and further perform a grid dependence test using the *k*-*ε* turbulence model. Shih *et al*.^35^ presented a new formulation for the *k*-*ε* eddy viscosity model, which was quite different from the standard *k*-*ε* model. The realizable model is based on a new dissipation rate equation and eddy viscosity formulation^36^. The governing equations of the realizable *k*-*ε* model, are presented below (Eq. (1) and Eq. (2)) and the modified eddy viscosity formulation may be referred to from the works of Shih *et al*.^35,36^. It is worthwhile to note that although the formulation for turbulent kinetic energy (*k*) remains identical to standard and RNG *k*-*ε* models, the difference lies in turbulence dissipation rate (*ε*) relationship. There is no generation of turbulent kinetic energy in the production term of turbulence dissipation rate formulation. In addition, the destruction term in denominator never vanishes thus negating the possibility of singularity even if the turbulent kinetic energy vanishes or becomes less than zero. The changes implemented in this eddy viscosity model lead to a better representation of spectral energy transfer. It was found that the realizable *k*-*ε* model performed better for separated flows as compared to standard *k*-*ε* and this fact provides motivation for its use in present study.

1 $$\frac{\partial }{\partial t}(\rho k)+\frac{\partial }{\partial {x}_{j}}(\rho k{u}_{j})=\frac{\partial }{\partial {x}_{j}}\left[\left(\mu +\frac{{\mu }_{t}}{{\sigma }_{k}}\right)\frac{\partial k}{\partial {x}_{j}}\right]+{G}_{k}+{G}_{b}-\rho \varepsilon -{Y}_{M}+{S}_{k}$$

2 $$\frac{\partial }{\partial t}(\rho \varepsilon )+\frac{\partial }{\partial {x}_{j}}(\rho \varepsilon {u}_{j})=\frac{\partial }{\partial {x}_{j}}\left[\left(\mu +\frac{{\mu }_{t}}{{\sigma }_{\varepsilon }}\right)\frac{\partial \varepsilon }{\partial {x}_{j}}\right]+\rho {C}_{1}{S}_{\varepsilon }-\rho {C}_{2}\frac{{\varepsilon }^{2}}{k+\sqrt{\nu \varepsilon }}+{C}_{1\varepsilon }\frac{\varepsilon }{k}{C}_{3\varepsilon }{G}_{b}+{S}_{\varepsilon }$$

where,

$${C}_{1}=\,\max \left[0.43,\frac{\eta }{\eta +5}\right]$$

$$\eta =\frac{k}{\varepsilon }\sqrt{2{S}_{ij}{S}_{ij}}$$

In the above equations, ${\sigma }_{k}$ and ${\sigma }_{\varepsilon }$ represent turbulent Prandtl numbers for $k$ and $\varepsilon$ respectively, while ${G}_{k}$ and ${G}_{b}$ are the generation of turbulent kinetic energy due to mean velocity gradient and the generation of turbulent kinetic energy due to buoyancy. While ${S}_{ij}$ represents mean strain rate, ${C}_{2}$ and ${C}_{1\varepsilon }$ are constants and, ${S}_{k}$ and ${S}_{\varepsilon }$ are user-defined source terms. Standard velocity inlet and pressure outlet boundary conditions were employed. Baseline tests were performed at length-based Reynolds number of 2.78 × 10^6^ and atmospheric pressure condition was imposed at the outlet. The no-slip condition was imposed on the walls of Ahmed body and road. Turbulent viscosity ratio was set to 10. Face values required for convection terms were assumed to be equal to cell centre values in the upstream cell for the first 100 iterations in each case, ensuring some stability in the solution. Post 100 iterations, all spatial terms in the cell-vertexed finite volume method were discretized using the second-order upwind scheme. A non-segregated pathway was followed for achieving the pressure-velocity coupling. Pressure correction equations and momentum equation are solved separately in the pressure-based segregated solver which results in slow convergence^37^. On the contrary, the pressure-based coupled algorithm, as used in this study, does a full discretization of the Rhie-Chow pressure dissipation terms as well as pressure gradient terms in the governing equations. Although a greater amount of memory was required for storing discrete momentum and pressure-based continuity terms, using the pressure-based coupled algorithm provided superior performance and reduced the number of iterations required for convergence significantly.

As suggested by Kelecy^38^, diagonal dominance of the coupled system is controlled by the CFL number, which is essentially less than 1 for explicit solvers and could be greater than 1 for implicit solvers. This number has a direct relationship with under relaxation of variables, also known as explicit relaxation, which governs the reduction in change in any variable produced between two successive iterations. In pressure-based coupled solver, the CFL number dictates to some extent, the rate of convergence and stability of the solution. Larger values enhance convergence, while smaller values enhance stability. CFL number used in this study is 60 with pressure and momentum explicit relaxation factors of 0.25 each, because of the complex physics involved. Under relaxation factors (URF) for turbulent kinetic energy, turbulent dissipation rate and turbulent viscosity were kept as 0.8 for the first 100 iterations post which, the URF for turbulent viscosity was changed to 0.98.

We chose three grids with approximately 1.8, 2.4 and 3 million elements for 25° slant case in the full domain and found a disagreement of about 0.3% between drag coefficients predicted by the fine and medium grids respectively, thus leading to the selection of a grid with 2.4 million elements. Moving on to the case of 35° slant, we chose four grids consisting of approximately 1.2, 2.2, 3.2 and 4.1 million elements and found a significant disagreement of about 1.2% between drag coefficients predicted by the medium and fine grids. The percentage difference between drag coefficient values predicted by latter of the two grids was 0.22%, thus leading to our choice of grid consisting of 3.2 million elements. As accounted for extensively in the literature, we found that the selected RANS model setup was able to predict the backlight separation zone for 35° slant quite effectively, but same was not true for 25° slant. Nevertheless, the ability of our numerical model to closely predict *C*_*D*_ values creates base for performing an investigation for validity of our innovative concept proposal. Using the solver parameters discussed above and grid sizing settings finalized upon, we performed a test case simulation on baseline cases of 25° and 35° of slant angles separately and compared the results with the experimental data of Lienhart and Becker^7^. *C*_*D*_ predicted by the chosen grid was within 4% and 4.8% of accuracy with experimental *C*_*D*_ for 25° case and 35° case respectively.

## Supplementary information

Supplementary information.
