# Supplementary material for: Towards a Novel Strategy for Safety, Stability and Driving Dynamics Enhancement During Cornering Manoeuvres in Motorsports Applications
Source: Sci Rep. 2020 Apr 14;10:6318. doi: 10.1038/s41598-020-63243-w (PMC7156714; doi:10.1038/s41598-020-63243-w)
Supplement: Supplementary file 1 — Supplementary information. [file 41598_2020_63243_MOESM1_ESM.pdf]

Towards a Novel Strategy for Safety, Stability and Driving Dynamics Enhancement During Cornering Manoeuvres in Motorsports Applications

Aditya Roy[1] and Debabrata Dasgupta*[1]

[1]Department of Mechanical Engineering, Indian Institute of Technology Delhi, New Delhi, India

Supplementary Information

1. Dependence of drag coefficient on Reynold's number

Meile et al. [14] showed a 13% decrease in C_D between $\mathrm{Re} = 0.7 \times 10^6$ and $\mathrm{Re} = 2.7 \times 10^6$ while Bayraktar et al. [5] reported a decrease of 3.5% in C_D between $\mathrm{Re} = 4.29 \times 10^6$ and $\mathrm{Re} = 13.2 \times 10^6$. The variation after $\mathrm{Re} = 9 \times 10^6$ was very less. This kind of trend in drag coefficients between 0.26 and 0.50 in commercial vehicles finds close correspondence with the report of Hucho[6]. From the variation of C_D of Ahmed body fitted with flaps with Re for both slant angles, as shown in figure S1, it may be observed that C_D shows a negligible decrement with increase in Re. There is a maximum of 1% decrement in C_D within the given range of Re, for a particular flap angle. A deeper investigation into pressure and viscous coefficients showed that while there was an increase in overall pressure drag, the decrease in overall viscous drag was dominant at higher Reynold's numbers. The ratio of pressure coefficient and viscous coefficient of Ahmed body is much greater than that of flap. Pressure drag in transcritical range shows a minor increase with Re due to flow separations and local changes in boundary layer state. On the other hand, skin friction or viscous drag shows a decrease with increase in Re. While viscous drag within the investigated Re range shows a sharp decrement in the case of simple Ahmed body due to transition of boundary layer from laminar to turbulent range because the role of skin friction decreases as we transition from laminar to turbulent flow, the same shows a rather less pronounced decrement in the cases with flap. The trend of variation is somewhat similar in both the slant angles. The 25° case is generally referred to as a high drag case and the 35° case is referred to as a low drag case. In spite of 25° slant being a high drag case, the maximum drag achieved is almost similar to the low drag case. There is about a 10% difference in C_D of low and high drag configurations for baseline case at $\mathrm{Re} = 2.78 \times 10^6$ while this difference comes down to a maximum of about 4% due to inclusion of side flap. Figure S2 shows the variation of lift coefficient (C_L) with Re for both the slant angles. 25° case is predominantly a high lift case and reverse is true for 35° case. The trend of variation between C_L and Re is quite similar to that between C_D and Re in a way that lift (positive or negative) generated on the vehicle geometry has minimal dependence on Reynold's number in transcritical range. In both the slant angles, amount of lift generated by flap angles of 70°, 80° and 90° is very similar. This can be made out from closeness of the bottom three curves in figure S2(a) and figure S2(b).

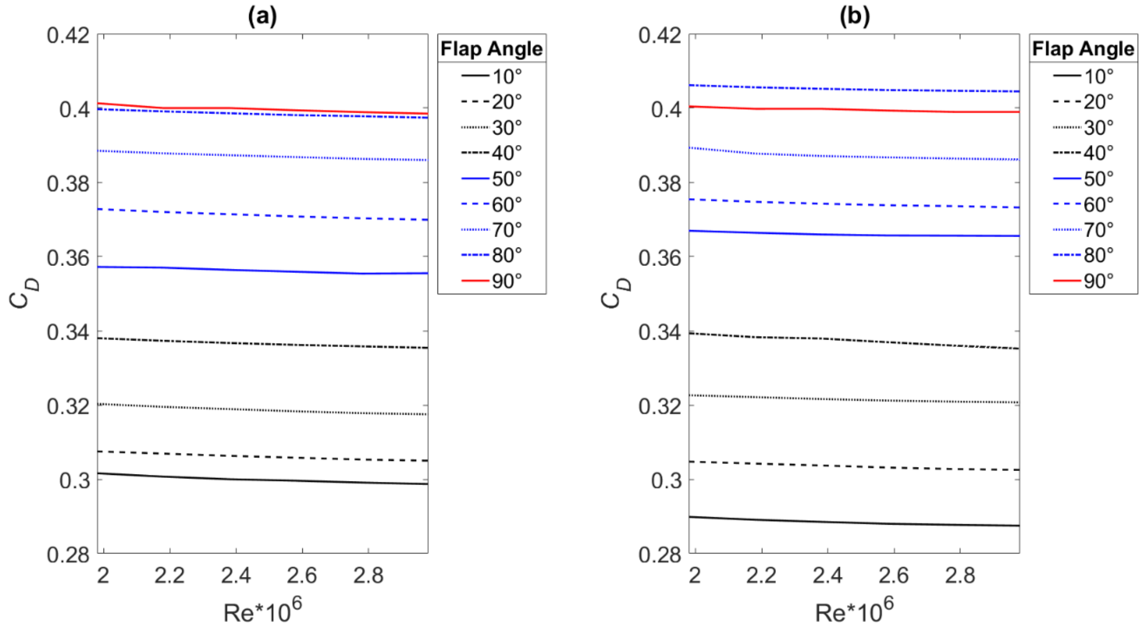

Figure S1. Variation of C_D with Re for (a) 25° and (b) 35° of slant angles.

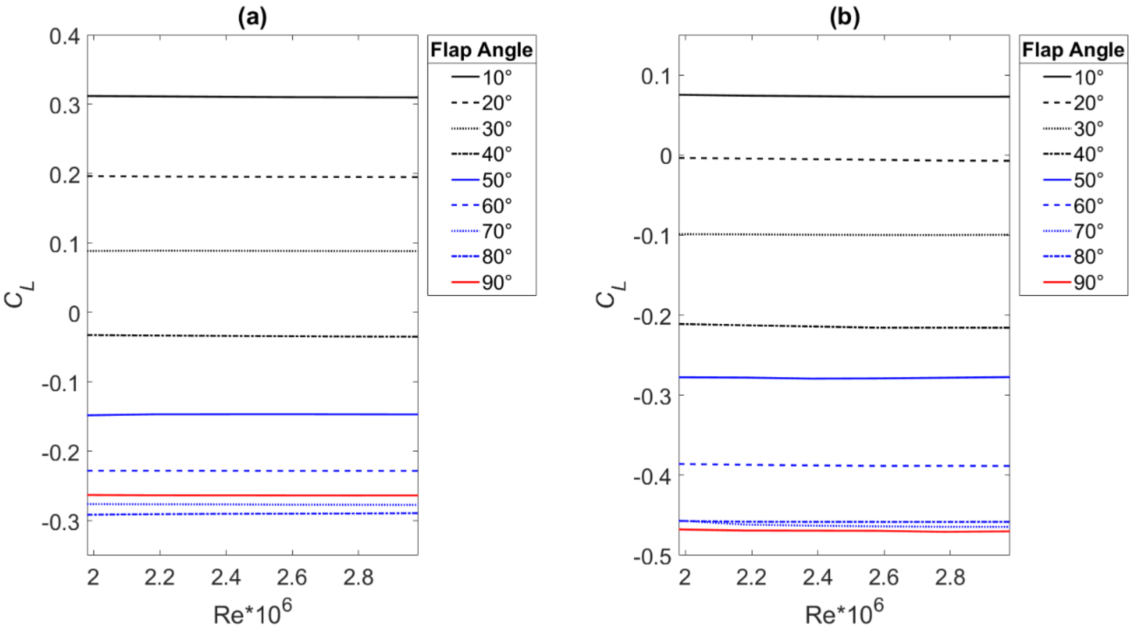

Figure S2. Variation of C_L with Re for (a) 25° and (b) 35° of slant angles.

2. Drag and downforce variations with flap angle

Changes in drag coefficient result in drag force variations, an account of which can be elucidated with the help of figure S3. A variation of percentage increase in drag force due to addition of side flap compared to baseline case, with flap angle is shown for both the cases of slant angles. The least amount of drag increment is exhibited by the configuration of 10° of flap angle at $\text{Re} = 1.98 \times 10^6$ in both the slant angles, their values being 5.5% and 9.1% for 25° and 35° slant respectively. Maximum drag increment equivalent to 70.6% and 87.1% for 25° and 35° cases respectively, is exhibited by the configuration of 90° and 80° flap angle, respectively at $\text{Re} = 2.98 \times 10^6$. It is evident that addition of side flap to an Ahmed body having 35° of slant angle exhibits a greater amount of drag force increment from its baseline case, thus resulting in higher wind resistance during turns and greater degree of decrement in braking effort required to slow down the vehicle. An important observation to be made is that of the drag variation above 80° of flap angle in both the slant angles. Figure S4 shows a variation of percentage increment in down force with flap angle for both the slant angles.

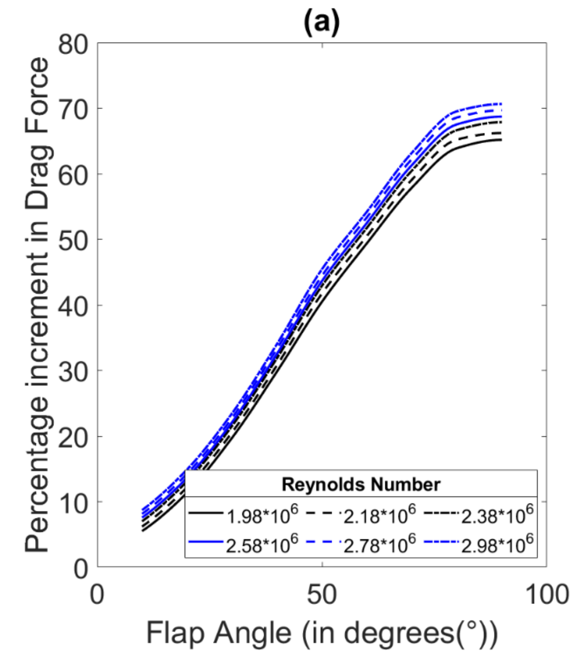

Figure S3. Variation of percentage increment in drag force with flap angle for (a) 25° and (b) 35° of slant angles.

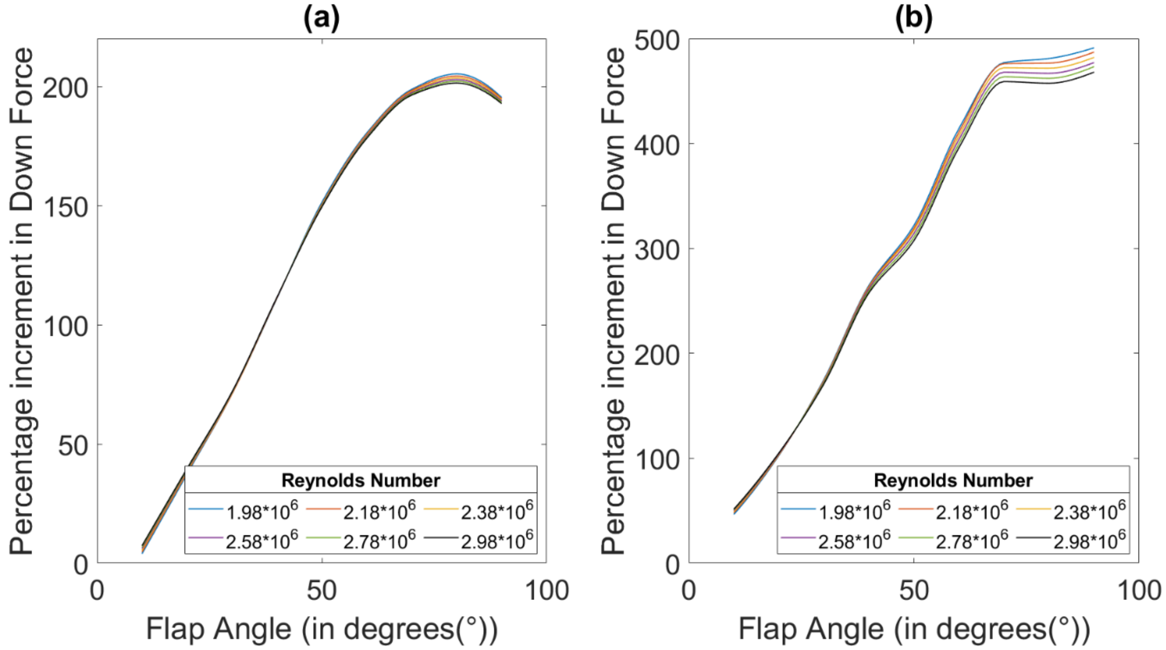

Figure S4. Variation of percentage increment in downforce with flap angle for (a) 25° and (b) 35° of slant angles.

3. Interaction of vortices

Figure S5 and figure S6 show velocity contours for the case of 50° flap angle at $Re = 2.78 \times 10^6$ plotted on the XZ plane at three different heights of 120 mm, 240 mm and 280 mm from the ground for 25° and 35° slant angle respectively. A comparison with baseline cases without flap is presented for reference. The vortex effects can be comprehended from low velocity regions in the contour. At a height of 120 mm, which is below the slant region, we can witness the under-developed flap-induced vortex (FIV). Drawing a comparison with wake region of the baseline case, we find that symmetricity of the same is disturbed as soon

as the FIV starts to build up. The wake region in 25° is more asymmetric than the 35° case (figure S6) at a height of 120 mm. As air flow moves past the whole span of the flap, effect of vortex generation gets more pronounced, as may be seen from contours mapped at 240 mm height. There is visible interaction of fluid flow between FIV and left-hand C-pillar vortex (LHCPV), and a significant difference between the left-hand and right-hand rear velocity distributions. The detachment zone over backlight is more prominent in the 35° case with larger recirculation zone. The plane at height of 280 mm shows how interaction of FIV and LHCPV increases as we move above the ground. The LHCPV is more pronounced at a height of 240 mm because it starts to develop at the beginning of the slant and gets fully developed at the end of backlight region. Proof of this fact is provided by a similar comparison of low velocity region at right-hand C-pillar vortex (RHCPV) on both the XZ planes (240 mm and 280 mm). On the other hand, the FIV is more pronounced at a height of 280 mm because it faces hindrance from a larger span of the flap by this time. This point of difference can be observed from the velocity contours at 240 mm and 280 mm height. A peculiar interaction of the two vortices generated at the left-hand rear region of the vehicle, as presented in figure S5 and figure S6 facilitate oversteer characteristics during negotiation of a right-hand turn and vice versa. Path lines indicating vortex interaction at the rear of the vehicle have been shown in figure S7 for both the slant angle cases.

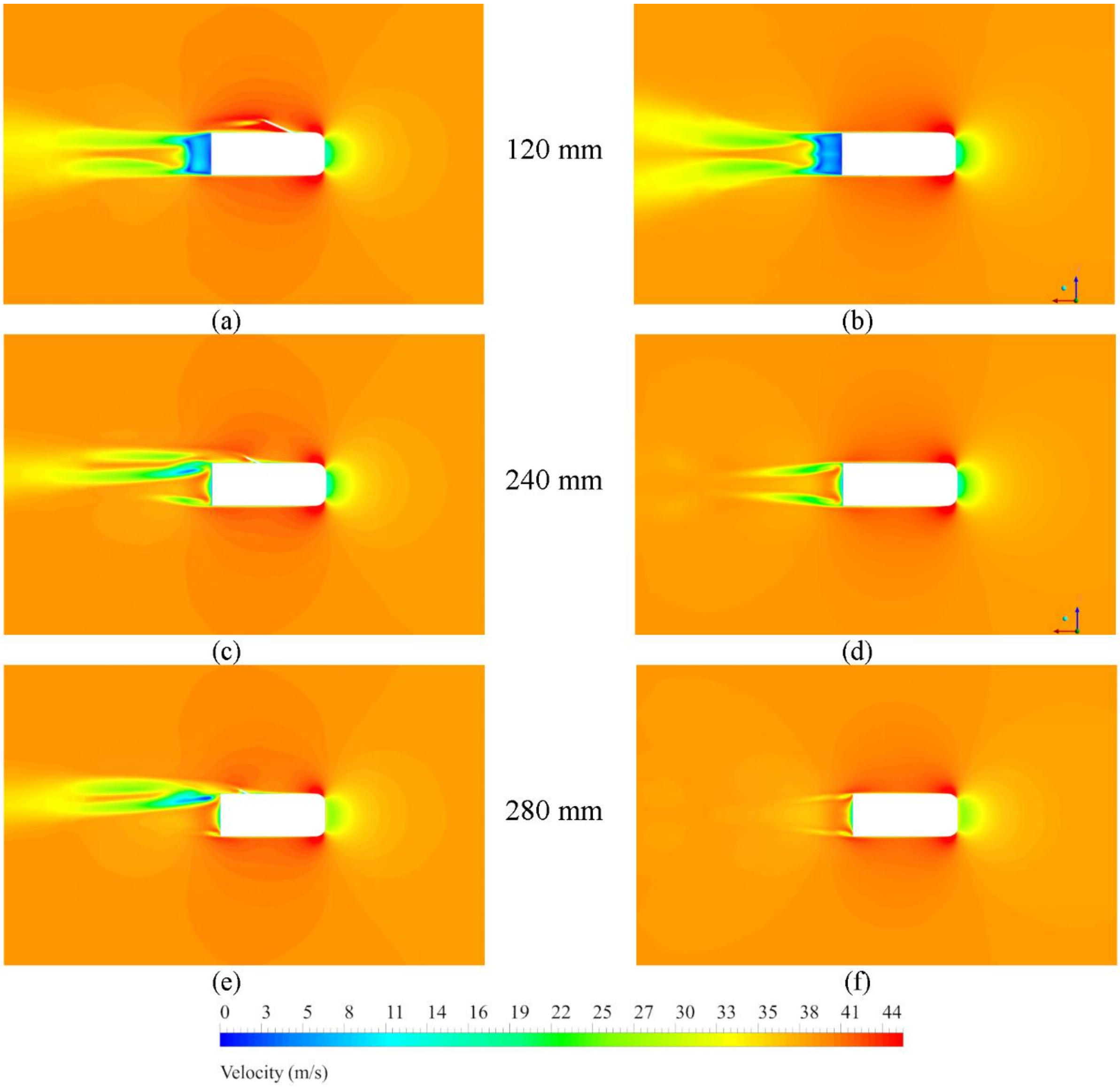

Figure S5. Velocity contours seen from the top of the vehicle having slant angle of 25° at indicated heights from the ground for (a),(c),(e) modified case with 50° of flap angle and (b),(d),(f) baseline case at Re $= 2.78 \times 10^6$.

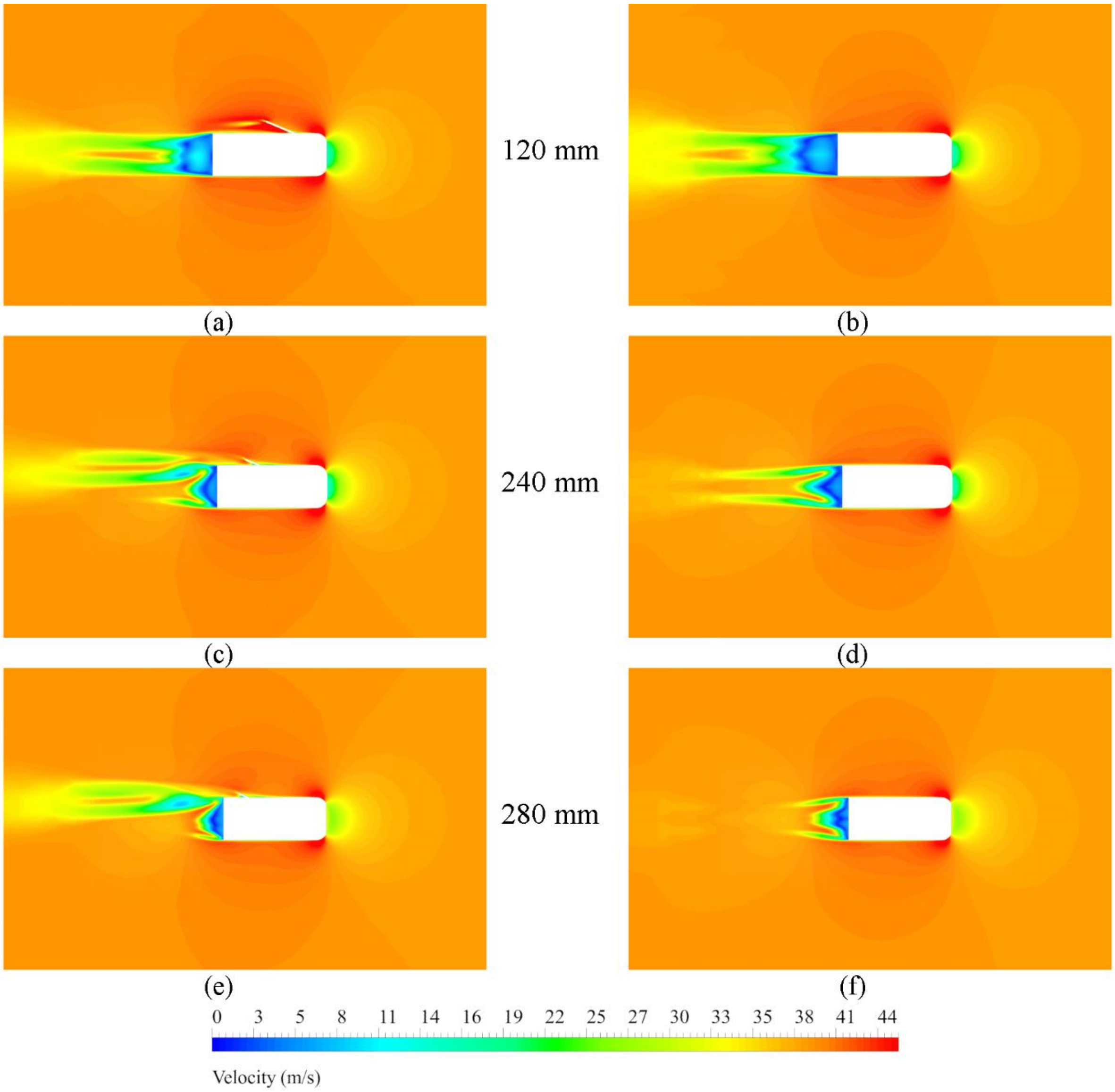

Figure S6. Velocity contours seen from the top of the vehicle having slant angle of 35° at indicated heights from the ground for (a),(c),(e) modified case with 50° of flap angle and (b),(d),(f) baseline case at Re $= 2.78 \times 10^6$.

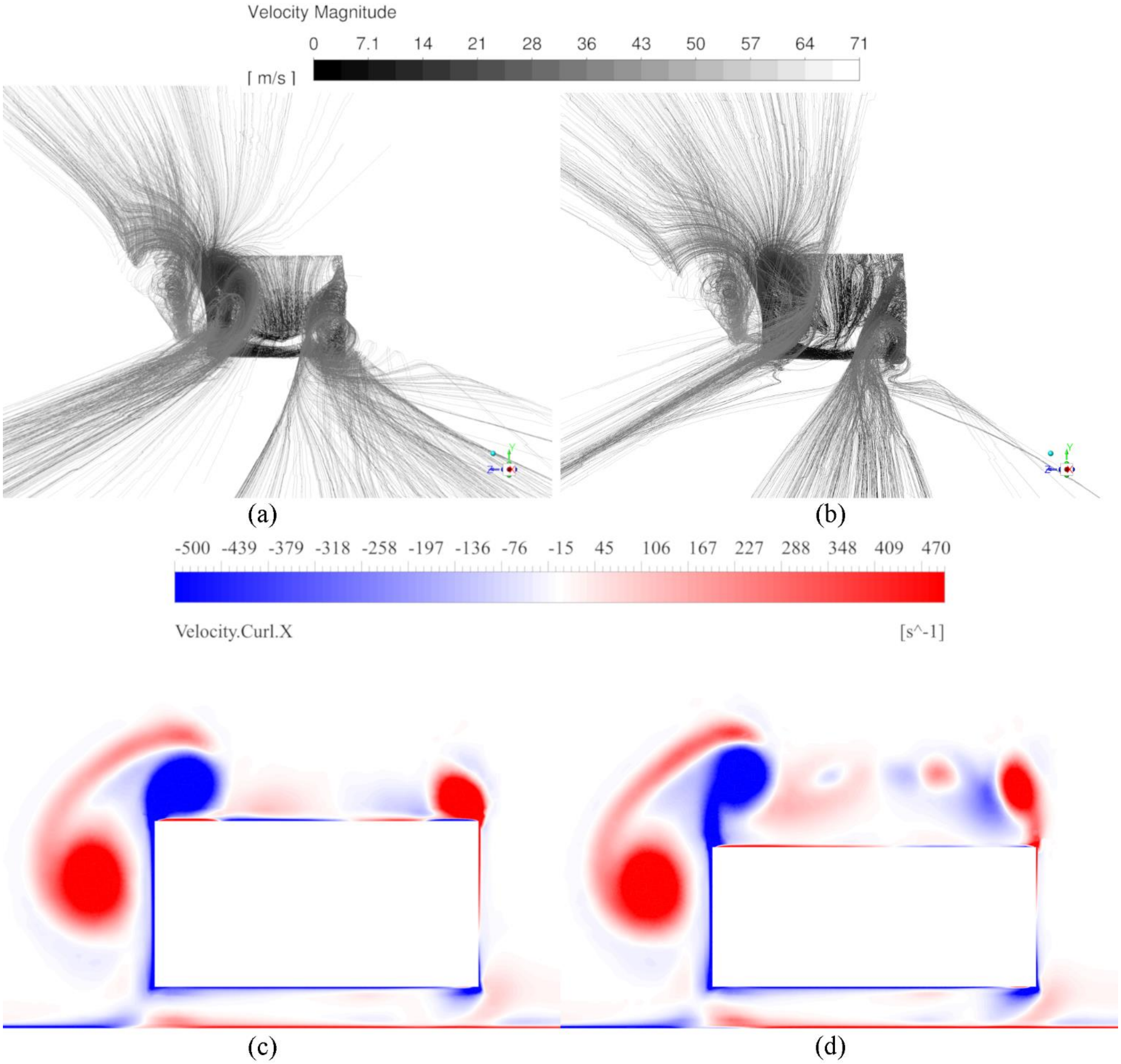

Figure S7. Path lines showing vortices at the rear for (a) 25° and (b) 35° slant configurations, and vorticity contours for (c) 25° and (d) 35° slant configurations with 50° of flap angle at Re $= 2.78 \times 10^{6}$.
